# One-Step Synthesis of Hexagonal Boron Nitrides, Their Crystallinity and Biodegradation

**DOI:** 10.3389/fbioe.2018.00083

**Published:** 2018-06-21

**Authors:** Özlem Şen, Melis Emanet, Mustafa Çulha

**Affiliations:** Department of Genetics and Bioengineering, Faculty of Engineering, Yeditepe University, Istanbul, Turkey

**Keywords:** hexagonal boron nitride, synthesis, biodegradation, boric acid, boron trioxide, colemanite

## Abstract

Hexagonal boron nitrides (hBNs) have recently been investigated for several novel applications due to their unique properties such as biocompatibility, superhydrophobicity, electrical insulation, and thermal and chemical stability. In addition, their biodegradation products have recently reported to have therapeutic effect on certain cancer types. hBNs are easily synthesized from boron and nitrogen precursors at moderately low temperatures. However, crystallinity and yield vary depending on the type of precursor, reaction temperature, and duration. In this study, a simple one-step hBNs synthesis method is reported without a catalyst, which might be an undesired contaminant for biomedical applications. The influence of boron precursors (boric acid, colemanite, or boron trioxide) on hBNs crystallinity, stability, and biodegradation in suspensions containing oxidative and hydrolytic degradation agents is investigated with the aim of their possible application in biomedicine. We found that the choice of boron precursor is a critically important parameter controlling the hBNs crystallinity and dependently influencing the biodegradation rate.

## Introduction

In recent years, boron nitride (BN) based nanomaterials have grasped attention of researchers for their possible use in medical applications due to their biocompatibility (Chen et al., 2009; Salvetti et al., 2015; Li et al., 2017), chemical and mechanical stability (Lahiri et al., 2011; Liu et al., 2016). Since they are composed of boron and nitrogen, they can be excellent needed as boron and nitrogen source in biosystems. Recently, it was demonstrated that BNs can be used as a boron source to promote wound healing and treat prostate cancer, and their degree of crystallinity is an important factor due to its influence on biodegradation (Dousset et al., 2002; Li et al., 2017).

Although hexagonal boron nitrides (hBNs) were discovered in 1842, their first stable form was obtained about a century later. The BNs do not naturally occur, thus they must be synthetically synthesized from boron and nitrogen precursors to prepare structures analog to their counterpart, graphene (Arenal and Lopez-Bezanilla, 2015). In hBNs, boron and nitrogen atoms are covalently bound in a hexagonal structure and their layers are stacked on top of each other. Similar to graphene, the two-dimensional (2D) BN layers interact with each other through van der Waals forces (Du Frane et al., 2016), and constitute hBN films (Shi et al., 2010) or spherical nanostructures (Bhimanapati et al., 2014), Given their excellent physical and chemical properties, hBNs are considered as promising nanomaterials in many technological applications (Han, 2010).

The use of hBNs in a range of applications was reported in the literature including their exploitation as 2D dielectric thin films and deep UV emitters at around 215 nm (Kubota et al., 2007; Song et al., 2010). It was also reported that composite of hBNs with polyether ether ketone showed improved mechanical and thermo mechanical properties, since hBNs possess high elastic modulus, excellent lubrication properties, and good thermal conductivity (Liu et al., 2016). In last few years, the hBNs are also gaining attention as a promising nanomaterial in medical and biomedical applications due to their high biocompatibility (Sukhorukova et al., 2015). Their use as a tablet lubricant in pharmacy and additive in cosmetics are also encouraging points for their consideration in the biomedical field (Turkoglu et al., 2005; Shi et al., 2010; Liu et al., 2016). In our recent study, their cytotoxicity was assessed, and conjugation with doxorubicin and folate has been proposed to enable their potential in cancer therapy (Emanet et al., 2017).

There are several hBNs synthesis methods reported in the literature by using a variety of boron and nitrogen precursors under different experimental conditions (Singhal et al., 2008). The synthesis methods influence reaction yield, number of layers, shape, and size of hBNs (Li et al., 2011). In one study, boric acid and urea were mixed and heated under ammonia atmosphere to produce hBNs (Chakrabartty and Kumar, 1995). In another, it was reported that hBN films were synthesized using diborane and ammonia as gas precursors, and the number of hBN layers can be controlled at various temperatures, gas pressure and flow rates (Ismach et al., 2012). Moreover, spherical BNs were synthesized using trimethoxyborane [B(OMe)_3_] under ammonia gas flow via chemical vapor deposition (CVD) method (Tang et al., 2008). It was found that the spherical morphology of the BNs strongly depends on the elimination yield of Me_2_O groups from the BN structures during the intermediate phase of the synthesis (Tang et al., 2008). In addition, hollow BN spheres were synthesized using B(OMe)_3_ and Ar instead of ammonia in the second stage of the synthesis that is the key point to form hollow structures. Furthermore, an increase in post-treatment temperature decreases the wall thickness of the hBNs while increasing the crystallinity of the structures (Li et al., 2017).

The studies reporting the inhibitory effect of boron containing compounds on cancer cell proliferation including prostate and breast cancer by reducing the release of stored Ca^2+^ ions into the cytosol of cells suggests that boron compounds can be promising agents for cancer treatment (Henderson et al., 2009). However, the frequent administration of boron compounds due to their short half-life for systemic administration limits their use because it requires continues administrations. Since boron-based nanomaterials such as hBNs can behave as a controlled boron release source (Li et al., 2017). Their use can open up new venues in cancer treatment. Therefore, it is important to synthesize hBNs as pure as possible with desired crystallinity, which might also be an important factor for further chemical modification with targeting ligands. These mentioned points could also be important factors for their dissolution/degradation in biological medium for a successful therapeutic approach (Barranco and Eckhert, 2004, 2006; Scorei et al., 2008; Emanet et al., 2015; Li et al., 2017).

In this study, from three different boron compounds, boric acid, colemanite, and boron trioxide were used to synthesize hBNs under ammonia gas atmosphere with CVD technique. The synthesized hBNs were characterized with imaging and spectroscopic techniques. The colloidal stability of the hBNs was investigated by monitoring zeta potential and time dependent size distribution with dynamic light scattering (DLS) technique. Thermogravimetric analysis (TGA) was carried out to observe the resistance against heat decomposition. Finally, the biodegradation behavior of the hBNs in oxidative and hydrolytic degradation conditions was assessed by TGA, ICP-MS, and Raman spectroscopy.

## Materials and methods

### hBNs synthesis

Boric acid, colemanite, and boron trioxide, as boron and ammonia as nitrogen source were used. The synthesis was performed based on a method previously reported by our group (Emanet et al., 2017). First, 2 g of boric acid or colemanite or boron trioxide were suspended in 3 mL of 13.38 M ammonia solution. This mixture was transferred onto a silicon carbide boat and dried on a hot plate adjusted to 100°C for approximately 20 min. Then, this silicon carbide boat was placed in a Protherm Furnace PTF 14/50/450 and heated until 1,300°C with a heating rate of 10°C/min under ammonia gas flow for 2 h. Following the heating, the silicon carbide boat was removed from furnace at around 550°C and hBNs were scratched from the surface of the silicon carbide boat with the help of spatula and stored under room conditions.

### SEM and TEM imaging

The morphology and size of the synthesized hBNs were characterized using SEM and TEM. SEM (Helios Nano-Lab 600i FIB/SEM, FEI) imaging was carried out on samples previously gold-sputtered for 25 s at 60 nA, obtaining a 3-nm thick conductive layer over the hBNs. TEM images were acquired with a JEOL-2100 HRTEM microscopy system at 200 kV (equipped with LaB6 filament and an Oxford Instruments 6498 EDS system).

### UV-Vis, XRD, FT-IR, and Raman spectroscopy

The hBNs were dispersed in double distilled water (ddH_2_O) by sonication for 2 min at before the analysis (Bandelin Sonopuls HD 3100). A Perkin Elmer Lambda 25 UV-Vis spectrometer was used to obtain absorption spectra. IR spectra were acquired with a Thermo NICOLET IS50 Spectrometer. XRD analysis was performed using a Shimadzu XRD-6000 with a ICDD PDF 4 software. The scanning area was in continuous mode with a scanning range of 2.000–69.980° and a scanning speed of 2.0000°/min. The sampling pitch was set to 0.0200°, and the preset time was set to 0.60 s. Raman spectra of the hBNs were recorded using a Renishaw In Via Reflex Raman Microscopy system (Renishaw Plc., New Mills, Wotton-under-Edge, UK) equipped with a 514 nm Argon ion laser. A minimum of 16 spectra was acquired from a 16-μm^2^ hBNs sample area. All measurements were performed at least three times.

### Thermogravimetric analysis (TGA)

TGA analyses were performed using a Mettler Toledo TGA/SDTA 851 instrument. The samples were analyzed under 20-mL/min N_2_ gas flow. The temperature was increased up to 700°C and was set at 10°C/min.

### Dynamic light scattering (DLS) and zeta potential measurements

The colloidal stability analysis of the hBNs was carried out by monitoring the size distribution and the Z-potential using a Malvern Zetasizer Nano ZS. The hBNs were sonicated for optimal dispersion in ddH_2_O at for 2 min. The concentration of the hBNs was fixed as 1 mg/mL. Then, the sonicated samples were analyzed using DLS at different times (0 and 60 min) to assess their time-dependent colloidal stability. Furthermore, zeta potential measurements were performed and all experiments were repeated at least three times.

### Biodegradation studies

Biodegradation of hBNs was performed under two different conditions: in lysosome mimicking solution (LMS) for oxidative degradation, and in phosphate-buffered saline (PBS) for hydrolytic degradation. To observe the oxidative degradation of hBNs in lysosome mimicking condition, a LMS was prepared according to Table 1 (Russier et al., 2011). The pH of the solution was brought to 4.5. Then, 25 mg of hBNs were dispersed in 25 mL of LMS, which contains physiological concentration of hydrogen peroxide (H_2_O_2_, 1 mM). Then, they were sonicated for 1 h. To maintain the physiological oxidizing environment of lysosomes, 1 mM of H_2_O_2_ was weekly added. For hydrolytic degradation of hBNs in PBS, 25 mg hBNs were dispersed in 25 mL PBS and sonicated for 1 h. After the sonication, all samples for oxidative and hydrolytic degradation were placed in dark and incubated at 37°C while shaking at 180 rpm up to 30 days. In both cases, 1 mL sample was taken from the LMS or PBS suspensions at 0, 1, 3, 7, 14, and 30 days of the incubation, and stored at 4°C in the dark until characterization.

**Table 1:** Composition of LMS.

**Composition of lysosome mimicking solution**	**Concentration**
Calcium chloride dehydrate	197.0 mM
Sodium chloride	113.8 mM
Potassium hydrogen phthalate	0.0 mM
Glycine	6.0 mM
Sodium phosphate dibasic anhydrous	1.0 mM
Sodium sulfate (anhydrous)	0.5 mM
Alkylbenzyldimethylammonium chloride	50.0 ppm

#### Characterization of degradation products

After the degradation process in the LMS and PBS suspensions, samples taken from the suspensions were used for TGA analysis. An unprocessed hBNs suspension was used as a control for comparison. The suspensions were centrifuged at 10,000 rpm for 10 min, the supernatants were removed, and the pellets including hBNs were dried under vacuum. The TGA analysis was carried out using the pellets.

The degradation products were evaluated with ICP-MS to determine boron content. First, 1 mL of the samples was centrifuged at 10,000 rpm for 10 min at 4°C to remove non-degraded hBNs from solution as a pellet. Then, 0.5 mL of the supernatant containing degradation products was mixed with 4.5 mL of 1% nitric acid solution. Thereafter, the samples were filtered with a 0.22 μm Millipore filter to further remove any non-degraded hBNs from the samples, which were evaluated with an X Series 2 ICP-MS (Thermo Scientific) instrument equipped with a CETAC asx-520 autosampler. In the ICP-MS system, the plasma power, the plasma gas, the nebulizer gas flow rate, the clutch duration, and the wash duration were set to 1,350 W, Ar, 0.95, 35, and 35 s, respectively. VHG Labs Z frequency 1007-100 multi-element standard stock solution (1 mg/mL) containing Al, B, Cu, Ag, As, Cd, Fe, Ni, Sr, Zn, Ca was used for calibration. The standards were prepared at the concentrations of 0.1, 1.0, 10.0, and 100.0 μg/mL in 5% nitric acid using this stock solution. The calibration curves were created for each metal and the experiments were performed at least three times.

The characterization of hBNs after the degradation process was further analyzed by Raman spectroscopy. Five microliters of LMS and PBS suspensions containing hBNs were placed on a CaF_2_ slide and allowed to dry. A minimum of 16 spectra was recorded from a dried droplet area by choosing arbitrary points. All experiments were performed at least three times and their average values were calculated.

## Results and discussion

### Morphological characterization of hBNs

**Figure 1:**
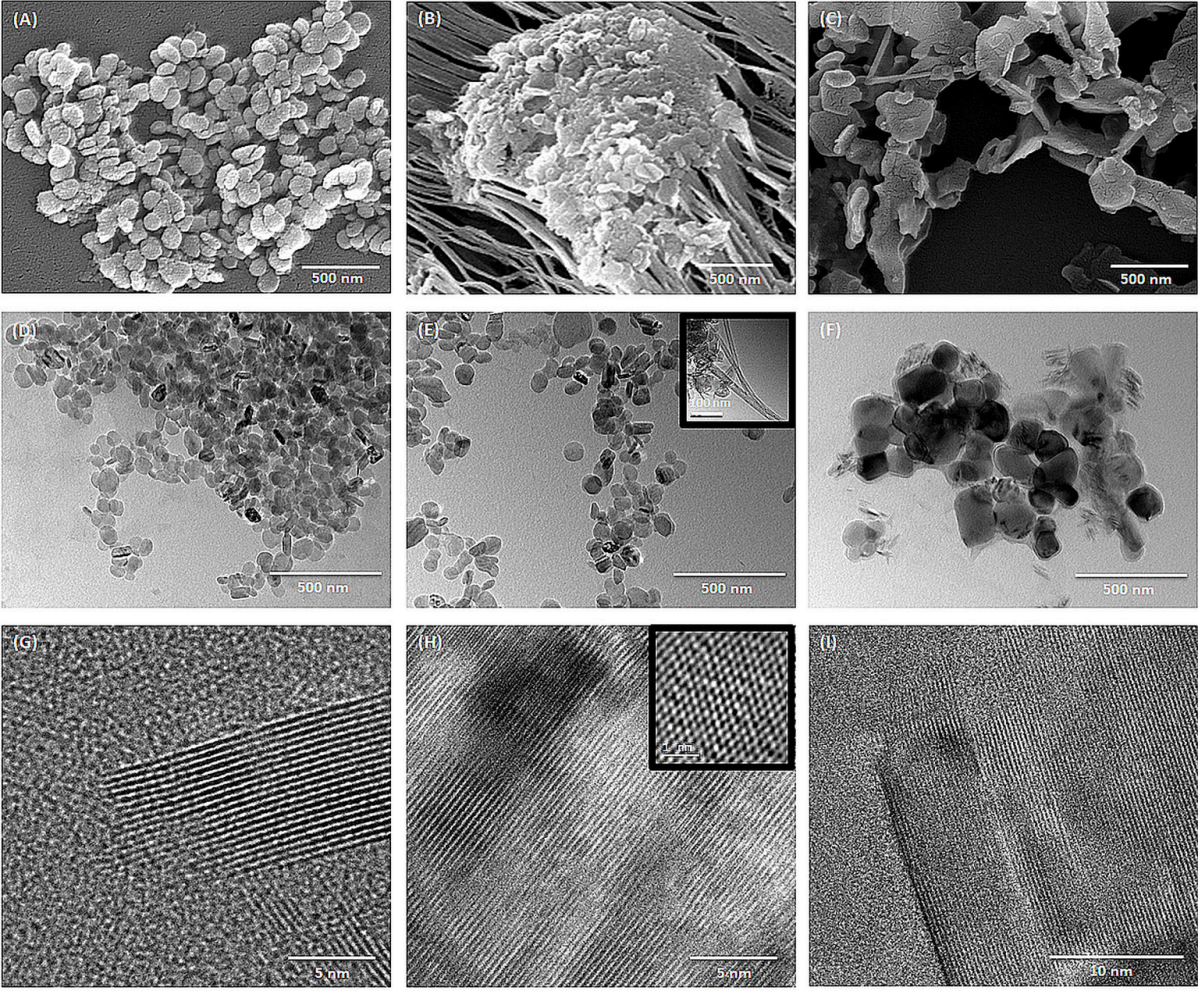
SEM images of **(A)** hBNs_boric acid, **(B)** hBNs_colemanite, and **(C)** hBNs_boron trioxide. TEM images of **(D,G)** hBNs_boric acid, **(E,H)** hBNs_colemanite, and **(F,I)** hBNs_boron trioxide.

The size and morphology of hBNs can vary depending on synthesis conditions and the type of boron precursor. Figure 1 shows the SEM and TEM images of the hBNs synthesized from three different boron precursors. As seen, the hBNs synthesized from each precursor have platelet-like morphology with varying sizes. The hBNs synthesized from boric acid (hBNs_boric acid) show a more uniform structure with lateral size dimension between 50 and 70 nm as seen in Figure 1A while the lateral size dimensions of the hBNs synthesized from colemanite (hBNs_colemanite) are between 50 and 80 nm. The hBNs_colemanite also include boron nitride nanotubes (BNNTs) in the product mixture (Figure 1B). This can be explained with the presence of metal oxides such as CaO, B_2_O_3_, Fe_2_O_3_, Al_2_O_3_, MgO, and Na_2_O in colemanite acting as catalysts (Gür, 2007). Transition metals and their oxides play an important role in synthesis of BNNTs as catalysts to roll the hBN layers into tube shape (Pakdel et al., 2012). The hBNs synthesized from boron trioxide (hBNs_boron trioxide) show non-uniform lateral sizes in the range of 200–300 nm indicating a poor regularity as seen in Figure 1C. Figures 1D–F show TEM images of the synthesized hBNs. As seen in Figure 1D, the hBNs_boric acid present uniform platelet-like structures while both hBNs and incomplete BNNTs are visible in the hBNs_colemanite sample reported in Figure 1E (inset image) similar to the case observed in SEM images. Figure 1F shows that the hBNs_boron trioxide have varying sizes confirming their structures as observed from their SEM images. Figures 1G–I show the parallel straight-line like crystalline features of the synthesized hBNs indicating the high quality of the samples. The honeycomb lattice structure of atomic boron and nitrogen was observed using HRTEM as shown in Figure 1H as inset image.

### Spectroscopic characterization of hBNs

**Figure 2:**
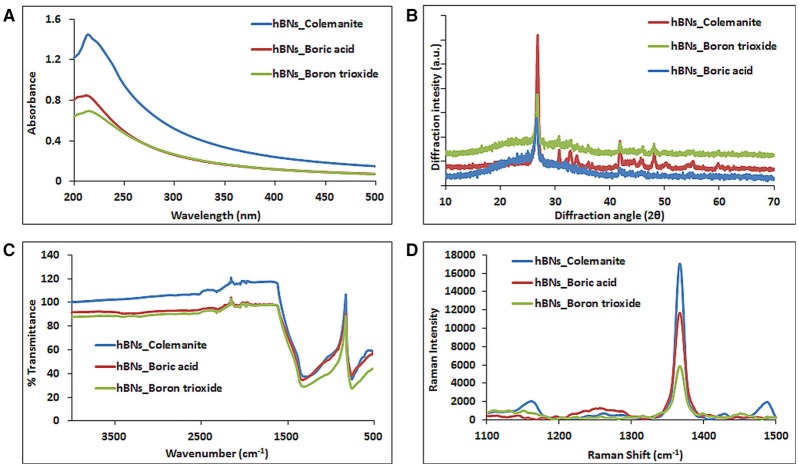
Characterization of hBNs obtained from various precursors. **(A)** UV-Vis, **(B)** XRD, **(C)** FT-IR, and **(D)** Raman spectroscopy.

The hBNs were characterized with UV-Vis, XRD, FT-IR, and Raman spectroscopy. Figure 2A shows comparison of UV-Vis spectra of hBNs synthesized from all three precursors. A characteristic maximum absorption band at around 210 nm is observed on the spectra. The highest absorption and background intensity are observed with the hBNs_colemanite probably due to the presence of BNNTs and other impurities in the final product (Behura et al., 2015).

Figure 2B shows the XRD pattern of the synthesized hBNs. The peaks originating from hBNs were observed at 2θ angles of 26.8° and 41.8° indicating successful synthesis for all hBNs derived from three different precursors. The synthesized hBNs show high crystallinity exhibiting narrow peak width around 26.8° and 41.8°. However, hBNs_colemanite show the highest crystallinity with respect to the hBNs_boric acid and hBNs_boron trioxide characterized with the increased peak intensities. Furthermore, hBNs_colemanite have varying peaks around 30–40° attributed to characteristic peaks of colemanite (Bayca et al., 2014).

The FT-IR spectra show that the hBNs have broad peaks at around 1,364 and 820 cm^−1^ that is attributed to the B–N vibrations as shown in Figure 2C. Moreover, a weak band at around 3,400 cm^−1^ attributed to O–H stretching in hBNs_boric acid and hBNs_boron trioxide, which is an important indicator for the degradation tendency of samples (Tang et al., 2008).

Figure 2D shows the Raman spectra of the hBNs with a sharp peak at around 1,364 cm^−1^ originating from B–N vibrations. The spectroscopic evaluation of the hBNs obtained from the boric acid, colemanite, and boron trioxide shows the unique characteristic spectral features for hBN structure.

### Colloidal stability of hBNs

Zeta potential of nanomaterials can be used as an indicator for their colloidal stability in aqueous environment. It is a fact that neutral and low density of charged molecular structures or nanomaterials cannot resist to attractive forces and form aggregates while highly charged particles repel each other and show better stability (Zhang et al., 2008). The zeta potentials of the synthesized hBNs in this study were measured and shown in Table 2. As seen, the zeta potential values are −20.72 ± 0.11 for the hBNs_boric acid, −18.02 ± 0.07 for the hBNs_colemanite, and −18.96 ± 0.08 for the hBNs_boron trioxide. These values indicate that the stability of colloidal suspensions of hBNs in aqueous environment is quite good.

**Table 2:** Zeta potential of synthesized hBNs.

**Samples**	**hBNs_boric acid**	**hBNs_colemanite**	**hBNs_boron trioxide**
Zeta potential (mV)	−20.72 ± 0.11	−18.07 ± 0.07	−18.96 ± 0.08

**Figure 3:**
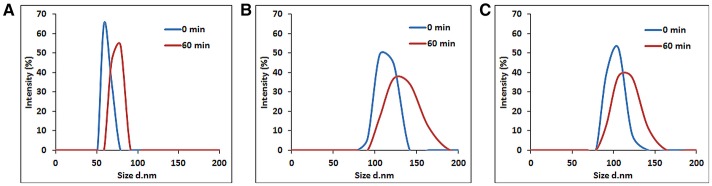
Size distribution of **(A)** hBNs_boric acid, **(B)** hBNs_colemanite, and **(C)** hBNs_boron trioxide.

In addition, their hydrodynamic stabilities were investigated by measuring their particle size distributions at different time-points from 0 to 60 min with DLS (Figure 3). The samples were sonicated for 2 min for an optimal dispersion. The size distribution of the hBNs_boric acid was found between 50 and 91 nm as shown in Figure 3A, and the maximum hydrodynamic size increased to 78 from 58 nm after 60 min. The size distribution of the hBNs_colemanite was found to be between 78 and 190 nm as shown in Figure 3B, and their maximum size was also approximately increased to 141 from 122 nm. The size distribution of hBNs_boron trioxide was between 78 and 164 nm as shown in Figure 3C, and their maximum size approximately increased to 122 from 105 nm after 60 min.

The results indicate that the hBNs_boric acid are more efficiently dispersed in aqueous environment considering their smaller average size as compared to the hBNs_colemanite and hBNs_boron trioxide. Furthermore, it has been observed that the size distribution of hBNs_boric acid is narrower as compared to the hBNs_colemanite and hBNs_boron trioxide indicating higher uniformity of hBNs_boric acid. Besides, the low differences in size distribution of each sample at 0 and 60 min show their high colloidal stability confirming the Z-potential results.

### Thermal stability of hBNs

**Figure 4:**
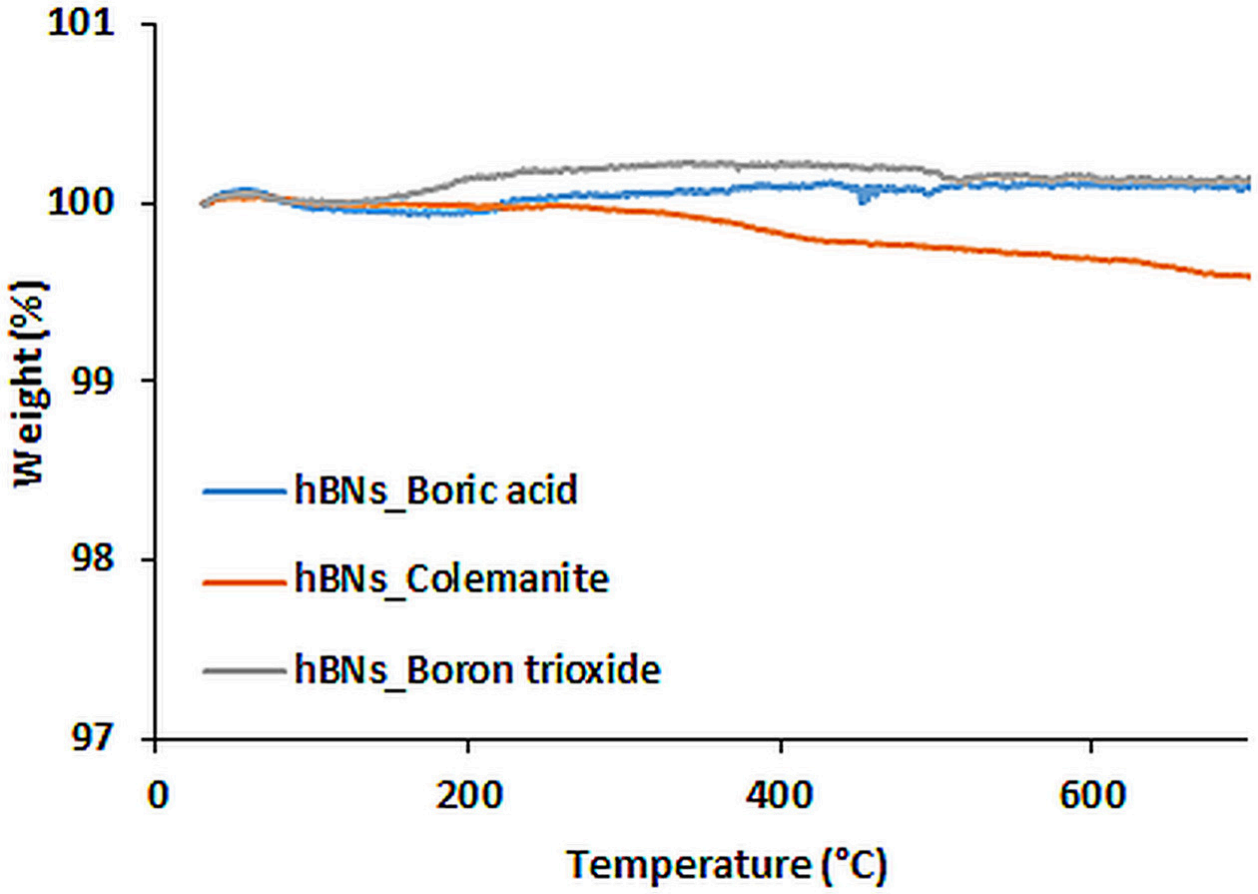
TGA curves of hBNs synthesized from different precursors.

TGA analysis was performed to investigate thermal stability of the hBNs within the temperatures range of 30–700°C. Figure 4 shows TGA analysis of the synthesized hBNs. As seen, hBNs synthesized from all precursors have high thermal stability since no significant weight loss was observed with heating up to 700°C. A slight different result is observed for the hBNs_colemanite. This may be due to presence of metal oxides originating from colemanite. Since the hBNs_colemanite may include incomplete BNNTs in the sample, the high thermal stability of BNNTs also contributes to the thermal stability of the mixture (Ferreira et al., 2015; Kalay et al., 2015, 2016).

### Biodegradation of hBNs

Biodegradability of nanomaterials is an important issue to be investigated before their use in medicine (Naahidi et al., 2013). In addition to the therapeutic effect, biodegradable nanomaterials have an additional advantage since they can be eliminated from the body through their degradation (Kalashnikova et al., 2015). Furthermore, boron compounds such as boric acid can be used in some cancer types as therapeutic agents as mentioned earlier (Barranco and Eckhert, 2004; Scorei et al., 2008). However, frequent administration is needed because of short half-life circulation and low bioavailability of boron compounds. Thus, slow release of boron derivatives is important for therapeutic applications particularly in a cancer treatment (Li et al., 2017). Boron exists as boric acid at the physiological pH and it is considered having the therapeutic effect (Barranco and Eckhert, 2006).

**Figure 5:**
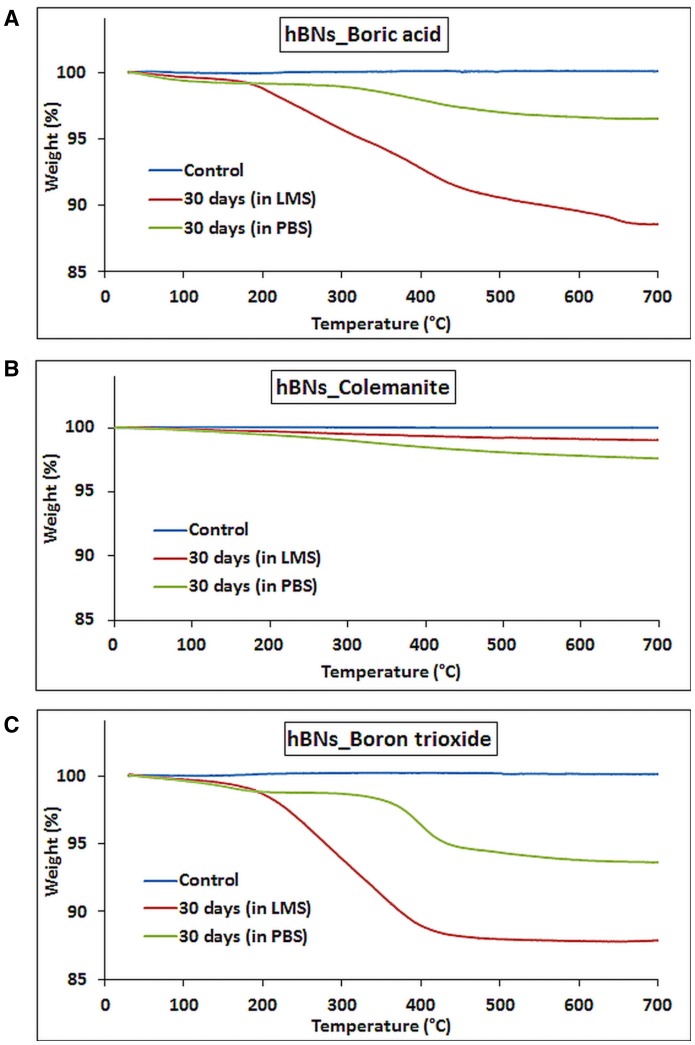
TGA curves of hBNs before and after biodegradation treatment. **(A)** hBNs_boric acid, **(B)** hBNs_colemanite, and **(C)** hBNs_boron trioxide.

The biodegradation of the hBNs under oxidative and hydrolytic conditions was examined for 30 days. After 30 days, hBNs in LMS or PBS suspensions were centrifuged to remove the supernatants. Then, TGA analysis was performed on the remaining pellets. Figure 5 shows the result of the experiments. The control hBNs_boric acid was found thermally stable during the temperature gradient. The approximate weight loss of hBNs_boric acid incubated in LMS was 12% while it was only 3% for hBNs_boric acid incubated in PBS. The mass loss of the hBNs_colemanite incubated in LMS was around 1% while it was 2% incubated in PBS indicating that hBNs_colemanite were chemically stable in LMS and PBS suspensions after a 30-day incubation as seen in Figure 5B. Furthermore, Figure 5C shows that approximately 12% weight of hBNs_boron trioxide incubated in LMS was lost while the mass loss of the hBNs incubated in PBS was found to be around 6%. The data show that hBNs_colemanite are more resistant to both oxidative and hydrolytic degradation processes compared to the hBNs synthesized from other two precursors. This might be again due to the presence of incomplete BNNTs along with the hBNs. In addition, the hBNs_boric acid and hBNs_boron trioxide were more resistant in hydrolytic degradation compared to the oxidative degradation. It is hypothesized that adding hydrogen peroxide into the test suspensions could damage the B-N bonds and these defects leads to the degradation of the hBNs. Furthermore, as seen in Figure 2C in FT-IR results, a weak band at around 3,400 cm^−1^, which is attributed to O–H stretching, could make the hBNs_boric acid and hBNs_boron trioxide more prone to oxidative degradation (Tang et al., 2008). The mass loss of the hBNs demonstrates that the degradation of the hBNs depends on their precursors and dependently crystallinity in both oxidative and hydrolytic degradation environments. Please note that after the centrifugation, it is possible that all hBNs are not precipitated as a pellet. However, the same experimental conditions were applied for all types of hBNs, we think that the trend in the data should be the same.

**Figure 6:**
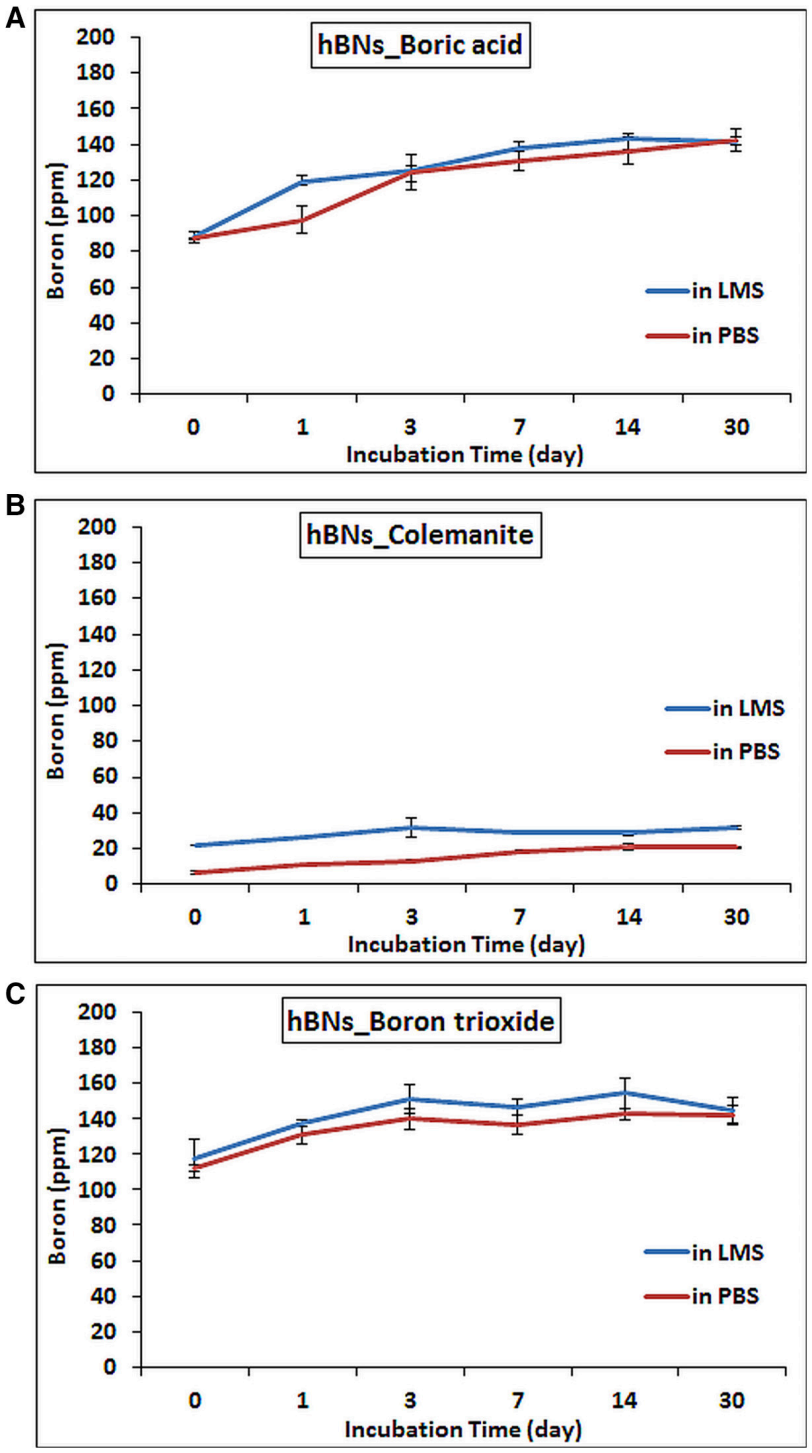
ICP-MS results of hBNs in LMS and PBS suspensions at increasing time points during biodegradation process. **(A)** hBNs_boric acid, **(B)** hBNs_colemanite, and **(C)** hBNs_boron trioxide.

The amount of the released boron from hBNs in the LMS and PBS suspensions along with their degradation is investigated using ICP-MS. It was found that as the incubation time increased, the boron content in their suspensions increased. After 30 days, the amount of released boron from hBNs_boric acid increased to 142.0 ± 6.4 ppm from 88.1 ± 3.0 ppm in LMS and PBS suspensions of hBNs_boric acid (Figure 6A). The boron concentration was found to be lower in both LMS and PBS suspensions of hBNs_colemanite as seen in Figure 6B, because of higher crystallinity of hBNs_colemanite. As mentioned earlier, crystallinity of the hBNs affects the boron release from the hBNs (Li et al., 2017). The amount of released boron from hBNs_colemanite increased to approximately 20.7 ± 0.4 ppm from 6.2 ± 0.9 ppm in PBS solution while the amount of released boron increased to approximately 31.4 ± 0.9 ppm from 22.0 ± 0.1 ppm in LMS. Thus, a decreased amount of boron release (approximately 7-fold) was found for hBNs_colemanite in PBS due to its crystallinity. After 30 days, the boron content in both suspensions of the hBNs_boron trioxide was found similar to the case of hBNs_boric acid as shown in Figure 6C. The amount of released boron from hBNs_boron trioxide increased to approximately 142.1 ± 5.4 ppm from 112.5 ± 1.8 ppm in PBS solution while the amount of released boron increased to approximately 144.5 ± 7.3 ppm from 117.2 ± 10.8 ppm in LMS.

**Figure 7:**
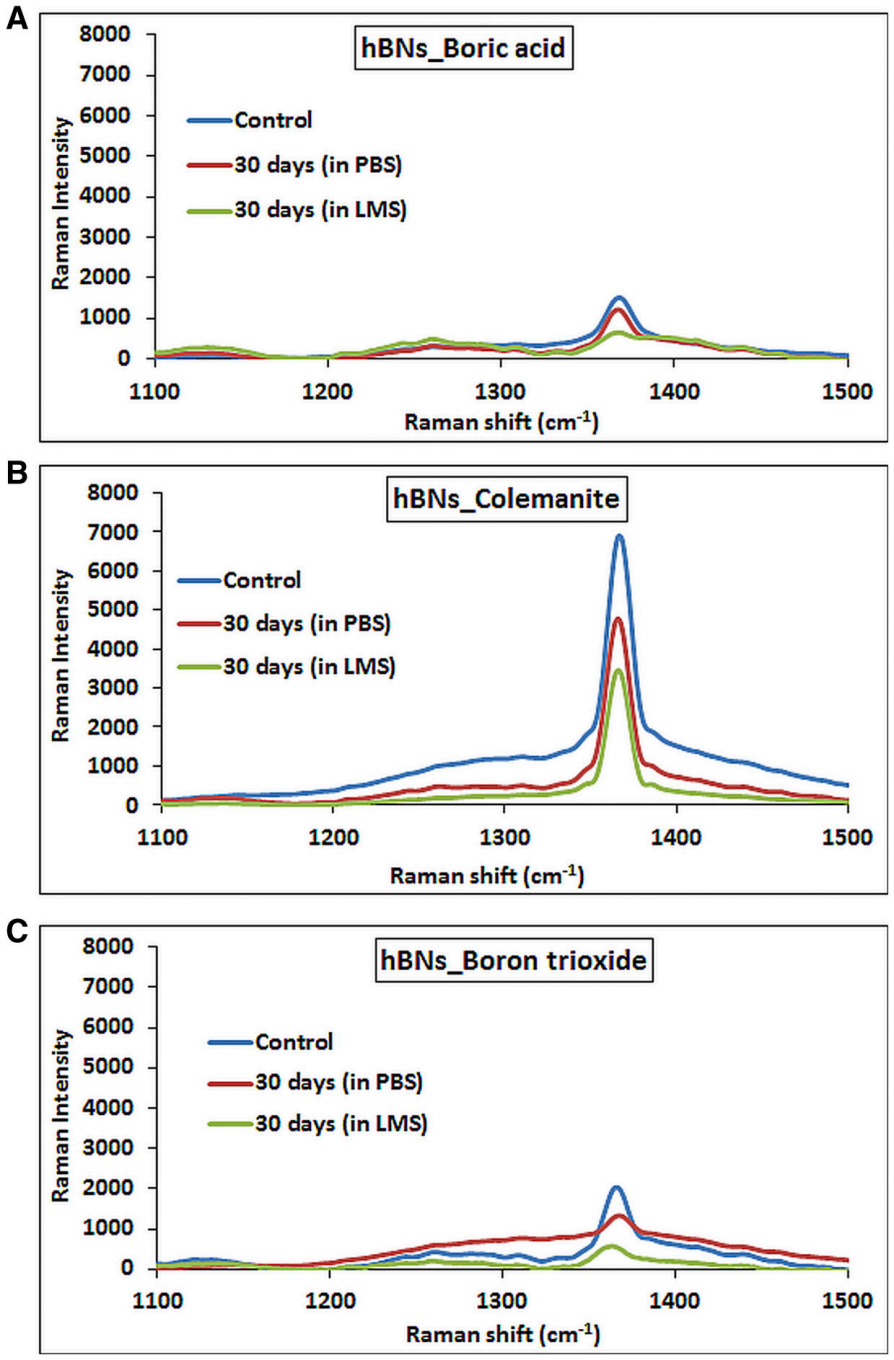
Raman spectra of hBNs before and after biodegradation process. **(A)** hBNs_boric acid, **(B)** hBNs_colemanite, and **(C)** hBNs_boron trioxide.

A further demonstration of hBNs degradation process was provided with Raman spectroscopy analysis. Figure 7A shows the Raman spectra of hBNs_boric acid before and after degradation process. The decrease of peak intensity at around 1,360 cm^−1^ suggests the degradation of the hBNs after 30-day incubation in LMS. For hBNs_colemanite, a sharp peak at around 1,360 cm^−1^ decreases as seen in Figure 7B resulting from the degradation of the hBN structures. The wide and decreased peak at around 1,360 cm^−1^ for hBNs_boron trioxide indicates the degradation of the hBNs after 30 days (Figure 7C). A decrease in the peak intensity at around 1,360 cm^−1^ demonstrates the biodegradation of the hBNs. These results are also in good agreement with TGA and ICP-MS results.

## Conclusion

In this study, hBNs were synthesized from three different precursors; boric acid, colemanite, and boron trioxide, and their crystallinity, size, shape, and biodegradation behavior were investigated. The morphological and spectroscopic characterization results indicate that the hBNs_boric acid show high crystallinity, uniform, and unique platelet-like structures. In the sample prepared from hBNs_colemanite, incomplete BNNTs were observed along with the highest crystalline hBNs. The formation of BNNTs is attributed to the presence of metal oxides behaving as catalysts. The hBNs_boron trioxide have also high crystallinity despite irregular lateral size dimensions. It is found that the size of the hBNs varies and their colloidal suspension is quite stable. The biodegradation studies show that hBNs_colemanite are more resistant to both oxidative and hydrolytic degradation (approximately 7-fold) due to their high crystallinity while hBNs_boric acid and hBNs_boron trioxide are more prone to both oxidative and hydrolytic degradation. The degradation study indicate a slow boron release possible in the form of borate, which can be an important finding in the treatment of certain cancer types (Barranco and Eckhert, 2006). The hBNs are suggested as possible therapeutic agents for use in some cancer types such as prostate due to boron content. In addition, using hBNs as a boron source might be a significant improvement for wound healing applications due their slow degradation. Over all, the type of precursors affects the synthesized product crystallinity, structural uniformity, and their biodegradation behavior. Thus, an appropriate boron precursor should be chosen based on the target application.

## Author contributions

ÖS and ME carried out the experimental work and the analysis of the data. All authors contributed to manuscript revision, read and approved the submitted version.

### Conflict of interest statement

The authors declare that the research was conducted in the absence of any commercial or financial relationships that could be construed as a potential conflict of interest.
